# Efficacy and safety of continuous antiviral therapy from preconception to prevent perinatal transmission of hepatitis B virus

**DOI:** 10.1038/s41598-020-70644-4

**Published:** 2020-08-12

**Authors:** Xingfei Pan, Jingsi Chen, Liyang Zhou, Xueting Ou, Fang He, Yifen Liu, Shuo Zheng, Haibin Wang, Bin Cao, Zhijian Wang, Huishu Liu, Guocheng Liu, Zhenyu Huang, Guanxin Shen, Shiliang Liu, Dunjin Chen

**Affiliations:** 1 Department of Infectious Diseases, The Third Affiliated Hospital of Guangzhou Medical University, Guangzhou, China; 2 Department of Fetal Medicine and Prenatal Diagnosis, The Third Affiliated Hospital of Guangzhou Medical University, Guangzhou, China; 3 Key Laboratory for Major Obstetric Diseases of Guangdong Province, Guangzhou, China; 4 The Medical Centre for Critical Pregnant Women in Guangzhou, Guangzhou, China; 5 Department of Obstetrics and Gynecology, The Third Affiliated Hospital of Guangzhou Medical University, No. 63 Duobao Road, Guangzhou, 510150 China; 6 Fujian Provincial Laboratory for Reproductive Health Research, School of Medicine, Xiamen University, Xiamen, China; 7 Department of Obstetrics and Gynecology, Nanfang Hospital, Southern Medical University, Guangzhou, China; 8 Department of Obstetrics and Gynecology, Guangzhou Women and Children’s Medical Center, Guangzhou, China; 9 Department of Obstetrics and Gynecology, Guangdong Provincial Maternal and Children’s Hospital, Guangzhou, China; 10 Department of Obstetrics and Gynecology, Beijing TsingHua Changgung Hospital, School of Clinical Medicine, Tsinghua University, Beijing, China; 11 Department of Immunology, Tongji Medical College, Huazhong University of Science and Technology, Wuhan, China; 12 School of Epidemiology and Public Health, University of Ottawa, Ottawa, Canada; 13 Centre for Surveillance and Applied Research, Public Health Agency of Canada, Ottawa, Canada

**Keywords:** Hepatitis B, Preventive medicine

## Abstract

Few studies were conducted to assess safety and efficacy of continuous antiviral therapy administrated from preconception. In the present study, 136 eligible women with chronic HBV infection were recruited, and assigned to active chronic hepatitis B (CHB) (Group A, B or C) or chronic HBV carrier (Group D). Antiviral therapy was administrated in preconception (Group A), in early (Group B) or late pregnancy (Group C and Group D). Immunoprophylaxis was administrated to all infants. Mothers’ HBV status and ALT were assessed at delivery and 7 months postpartum. Offspring’s HBV status was examined at 7 months old. Group A women showed low HBV DNA level and normal ALT throughout pregnancy. All women at delivery had an HBV DNA level of less than 10^6^ IU/ml, but the proportion of patients with lower HBV DNA level in Group A was higher than any of other three groups (*P* < 0.05). No differences in obstetrical complications were found among the four groups. None of infants who completed follow-up showed positive HBsAg at age of 7 months. Congenital malformation and infant growth indicators were similar among study cohorts. Continuous antiviral therapy from preconception to entire pregnancy is effective and safe for active CHB mothers and their infants.

## Introduction

Chronic HBV infection, a major cause for cirrhosis and hepatocellular carcinoma (HCC), remains an important public health issue worldwide, particularly in Asia and Africa^1^. Approximately 3.6% of the global population are chronically infected with HBV through perinatal transmission, a leading HBV transmission route^2^. Approximately 2 million children are newly infected with HBV every year through mother-to-child transmission (MTCT)^3^. Unfortunately, 90% of these children become chronic HBV infection that may possibly develop into progressive liver fibrosis, cirrhosis, even HCC in years or decades^4^. Therefore, blocking MTCT is a vital measure in reducing overall HBV prevalence and health burden.

The combined immunization of hepatitis B vaccine and hepatitis B immunoglobulin (HBIG) is generally used to prevent MTCT of HBV^4^. However, immunoprophylaxis fails in about 2–10% of infants^5,6^, whose mothers are with hepatitis B e antigen (HBeAg) positive or high viremia^7,8^. The failure rate can even increase to 30% for infants born to mothers with high HBV DNA level (HBV DNA ≥ 10^6^ IU/ml)^9–11^. Therefore, controlling maternal HBV DNA level is a critical measure for preventing MTCT of HBV. For mothers with high HBV DNA level, use of telbivudine (LDT)^12^ or tenofovir disoproxil fumarate (TDF)^13^ during early or late pregnancy to suppress viral replication, in combination with immunoprophylaxis, is considered to be an important approach to reducing MTCT of HBV^12–14^. However, some infants born to mothers undergoing antiviral therapy during the second or third trimester of pregnancy were infected with HBV^15^, suggesting that antiviral prophylaxis should start earlier. However, the best or optimal time for starting antiviral treatment remains uncertain.

Some child-bearing women with active chronic hepatitis B (CHB) should take oral antiviral drugs in preconception, because antiviral treatment for chronic HBV infection could control hepatitis flare and reduce the incidence of cirrhosis, HCC, and death^16,17^. These patients have to take oral antiviral drugs for a long time period, even lifelong, because oral antiviral drugs only inhibit HBV rather than kill HBV. However, hepatitis flare and virological recurrence are common after discontinuity of antiviral treatment^18,19^. Therefore, a long-term treatment is indispensable to maintain a virological response. Furthermore, HBV can be vertically transmitted to the offspring via germ cells^20^. It remains unclear whether the patients should continuously take antiviral drugs throughout pregnancy when their liver enzyme turns to be normal prior to becoming pregnant. To date, antiviral therapy is usually initiated after a child-bearing woman becomes pregnant. Few clinical studies about antiviral therapy were carried out during the embryo implantation phase. In the present study, we assessed whether continuous antiviral therapy initiated from preconception through the entire pregnancy is safe and effective to reduce MTCT of HBV.

## Results

**Figure 1:**
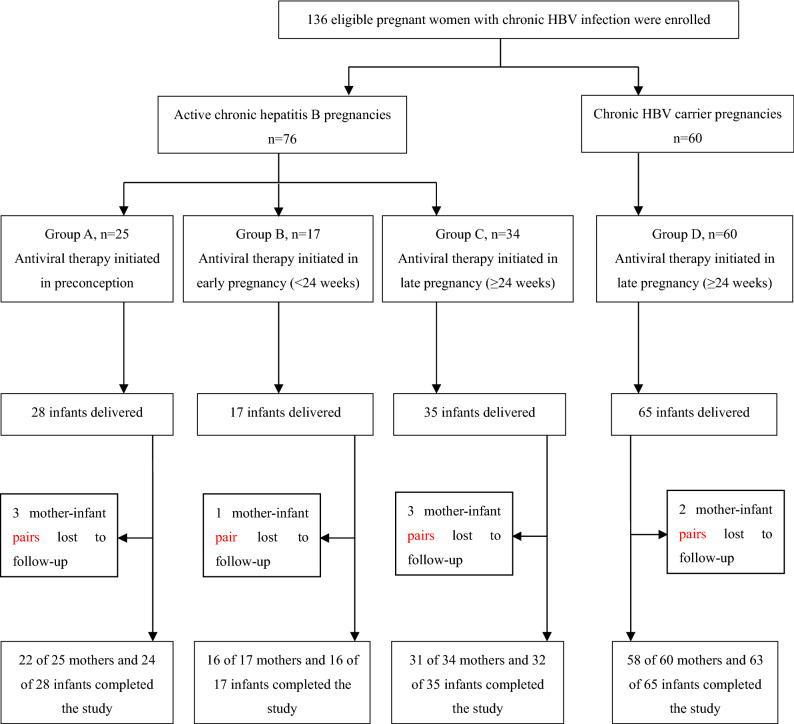
Enrollment and follow up of the study cohorts. Antiviral therapy means that TDF or LDT is used to treat the enrolled patients. *TDF* tenofovir disoproxil fumarate, *LDT* telbivudine, *HBV* hepatitis B virus.

From January 1st, 2017 to December 31st 2018, a total of 164 pregnant women were screened. A total of 136 eligible pregnant women were then enrolled and assigned to active chronic hepatitis B (CHB) groups (i.e., Groups A, B, or C) or chronic HBV carrier group (Group D). The antiviral therapy was administrated in preconception (Group A, 25 cases), in early (Group B, 17 cases) or late pregnancy (Group C, 34 cases and Group D, 60 cases), respectively (Fig. 1). Finally, we obtained the successful follow-up rates ranging from 85.7–96.9% for those four study groups.

### Efficacy and safety of antiviral therapy for mother

As shown in Table 1, there was no statistically significant difference in maternal age among these groups. In each group, the majority of enrolled cases were positive for hepatitis B e antigen (HBeAg). Group A patients showed a low HBV DNA level and normal ALT throughout pregnancy due to their initiation of antiviral treatment during preconception and continuity of the regimen throughout pregnancy. At delivery, the proportion of patients with low HBV DNA level in Group A was higher than any of other three groups (*P* < 0.05). Furthermore, all pregnant women in the four study groups had an HBV DNA level of less than 10^6^ IU/ml at delivery. Three of 17(17.6%) patients in Group B, 10 of 34 (29.4%) in Group C and 4 of 60 (6.7%) in Group D presented with abnormal ALT at delivery, but all Group A patients showed normal ALT at delivery.

**Table 1:** Maternal demographic information and clinical characteristics.

	Group A n = 25	Group B n = 17	Group C n = 34	Group D n = 60	χ2 or F	*P*
**Maternal demographic information**						
Age, (years), mean ± SD	30.6 ± 3.4	30.4 ± 4.5	31.9 ± 4.0	30.1 ± 4.8	1.228	0.302
TDF, n (%)	17 (68)	14 (82.4)	25 (73.5)	28 (46.7)		
LDT, n (%)	8 (32)	3 (17.6)	9 (26.5)	32 (53.3)		
HBeAg positive, n (%)	21 (84)	13 (76.5)	20 (58.9)	56 (93.3)		
**Maternal serum HBV DNA level at enrollment**						
HBV DNA level, log_10_ IU/ml, mean ± SD	< 2	6.8 ± 1.0	6.5 ± 1.1	7.2 ± 0.9	6.668	0.002
**Maternal serum HBV DNA level at delivery**						
HBV DNA level, log_10_ IU/ml, mean ± SD	< 2	2.4 ± 1.2	3.3 ± 1.1	3.3 ± 0.9	5.083	0.008
HBV DNA level < 10^6^ IU/ml, n (%)	25(100)	16(94.1)	31(91.2)	57(95.0)	90.235	< 0.0001
Undetectable HBV DNA level < 10^2^ IU/ml, n (%)	22(88.0)	7(41.2)	6(17.6)	9(15.0)	107.765	< 0.0001
**Maternal serum HBV DNA level postpartum 1–7 months**						
HBV DNA level, log_10_ IU/ml, mean ± SD	< 2	< 2	< 2	7.3 ± 0.7		
**Maternal serum ALT level at enrollment, delivery and postpartum 1–7 months (U/liter)**						
Enrollment, mean ± SD	28.6 ± 34.2	282.1 ± 193.9	169.9 ± 138.6	19.2 ± 11.0	40.357	< 0.0001
Before delivery, mean ± SD	14.3 ± 4.9	29.4 ± 16.8	35.3 ± 28.5	22.7 ± 30.4	3.088	0.030
Postpartum 1–7 months, mean ± SD	32.2 ± 37.8	21.5 ± 11.3	31.5 ± 34.5	69.4 ± 99.9	1.728	0.170

*TDF* tenofovir disoproxil fumarate, *LDT* telbivudine, *HBeAg* hepatitis B virus e antigen.

There were no statistically significant differences in delivery mode and gestational age among the four groups (Table 2). There were also no statistically significant differences in selected complications of pregnancy among the four study groups (*P* > 0.05).

**Table 2:** Maternal condition and perinatal outcome among antiviral therapy groups.

	Group A n = 25	Group B n = 17	Group C n = 34	Group D n = 60	χ2 or F value	*P*
Delivery by means of cesarean section, n (%)	15 (60)	7 (41.2)	17 (50)	25 (41.7)	2.735	0.434
Gestational age(weeks), mean ± SD	39.0 ± 1.9	39.1 ± 0.7	38.2 ± 1.5	38.9 ± 1.4	2.183	0.093
Pregnancy-induced hypertension, n (%)	2 (8.0)	0	1 (2.9)	3 (5.0)	1.637	0.651
Gestational diabetes, n (%)	4 (16.0)	0	7 (20.6)	8 (13.3)	4.306	0.230
Postpartum hemorrhage, n (%)	1 (4.0)	0	2 (5.9)	3 (5.0)	1.637	0.651
Preterm delivery, n (%)	3 (12.0)	0	4 (11.8)	2 (3.3)	6.029	0.110
Premature rupture of membranes, n (%)	4 (16.0)	2 (11.8)	6 (17.6)	9 (15.0)	1.876	0.599
Stillbirth, n (%)	0	0	0	0		
Maternal death, n (%)	0	0	0	0		

### Safety of antiviral therapy for fetus and infant

As shown in Table 3, there were no statistically significant differences in fetal development indicators and infants’ growth for the four groups (*P* > 0.05). Birth weight, body length and Apgar scores at 1 min were similar among the four groups (*P* > 0.05). There were no statistically significant differences in neonatal complications including congenital malformations, preterm birth and fetal/neonatal mortality (*P* > 0.05). There were no statistically significant differences in infants’ weight, length, abnormal development at age of 6 months among the four groups (*P* > 0.05).

**Table 3:** Basic information and fetal development indicators of offspring among antiviral therapy groups.

	Group A n = 28	Group B n = 17	Group C n = 35	Group D n = 65	χ2 or F value	*P*
**Basic information of infants**						
Twin, n	3	0	1	5		
Gender, female, n (%)	11 (39.3)	8 (47.1)	13 (37.1)	28 (43.1)	0.613	0.887
Birth weight (g), mean ± SD	3,409.1 ± 825.4	3,134.7 ± 288.3	3,103.3 ± 573.3	3,157.5 ± 628.3	1.389	0.249
Birth length (cm), mean ± SD	49.4 ± 2.6	50.1 ± 1.6	49.2 ± 1.7	49.2 ± 2.3	0.729	0.536
**Time from birth to administration of immunoprophylaxis (hour)**						
HBV vaccine, mean ± SD	11.3 ± 10.8	11.7 ± 9.0	9.2 ± 8.1	8.5 ± 6.7	0.956	0.416
HBIG, mean ± SD	11.4 ± 10.6	11.4 ± 9.3	9.2 ± 8.1	8.5 ± 6.7	0.945	0.422
**Complications of infants**						
Malformation, n (%)	0	0	1 (2.9)	0	2.917	0.405
Preterm birth, n (%)	3 (10.7)	0	4 (11.4)	2 (3.1)	6.679	0.083
Apgar score at 1 min, median (range)	9–10	9–10	8–10	10		
Newborn death, n	0	0	0	0		
**The development of infants at age 6 months**^**a**^						
	**n = 24**	**n = 16**	**n = 32**	**n = 63**		
Infant weight (g), mean ± SD	8,461.7 ± 962.1	8,060.6 ± 895.9	8,031.9 ± 1,214.4	8,092.6 ± 946.1	0.992	0.399
Infant length (cm), mean ± SD	69.7 ± 2.5	68.5 ± 2.5	68.7 ± 2.9	69.4 ± 3.1	0.974	0.407
Abnormal development, n	0	0	0	0		

*HBV* hepatitis B virus, *HBIG* hepatitis B immune globulin.

^a^4 infants of Group A, 1 infant of Group B, 3 infants of Group C, and 2 infants of Group D lost to follow-up.

### Effectiveness of antiviral therapy on MTCT of HBV

The MTCT rates of HBV for the four follow-up groups were shown in Table 4. None of infants who underwent the follow-up was positive for hepatitis B surface antigen (HBsAg) while all infants showed positive hepatitis B surface antibody (HBsAb). Group B showed the highest positive rate of antibody to hepatitis virus B core antigen (HBcAb), Group D showed the lowest (*P* < 0.05). Group A showed the lowest positive rate of HBcAb among the three active CHB groups.

**Table 4:** Infants’ HBV status and perinatal transmission outcomes of the study cohorts.

	Group A n = 24	Group B n = 16	Group C n = 32	Group D n = 63	χ2 value	*P*
**Infants’ HBV status at age 7 months**						
HBsAg positive, n	0	0	0	0		
HBsAb positive, n (%)*	24 (100.0)	16 (100.0)	32 (100.0)	63 (100.0)	4.122	0.208
HBeAg positive, n	0	0	0	0		
HBeAb positive, n (%)	0	0	3 (9.4)	3 (4.8)	4.994	0.172
HBcAb positive, n (%)	8 (33.3)	9 (56.3)	13 (40.6)	16 (25.4)	6.232	0.101
**Infants’ HBsAb value**						
≥ 1,000 mIU/ml, n (%)	8 (33.3)	4 (25.0)	6 (18.8)	17 (27.0)	2.506	0.474
100–999 mIU/ml, n (%)	4 (16.7)	6 (37.5)	4 (12.5)	11 (17.5)	1.245	0.742
10–99 mIU/ml, n (%)	0	1 (6.3)	0	7 (11.1)	7.150	0.067

*HBsAg* hepatitis B virus surface antigen, *HBsAb* antibody to hepatitis B virus surface antigen, *HBeAg* hepatitis B virus e antigen, *HBeAb* antibody to hepatitis B virus e antigen, *HBcAb* antibody to hepatitis B virus core antigen.

## Discussion

In the present study, we found that Group A patients showed low HBV DNA level and normal ALT throughout pregnancy. The proportion of patients with lower HBV DNA level in Group A was higher than any of other three groups (*P* < 0.05). At delivery, all Group A patients were with normal ALT, but some patients of other three groups were with abnormal ALT. Compared with patients who took antiviral drugs from late pregnancy (Groups C and D), patients who took antiviral drugs from preconception or early pregnancy (Groups A and B) did not show difference in obstetrical complications. Furthermore, their congenital malformation and neonatal growth indicators were similar among four groups. It is already confirmed that antiviral therapy initiated in the second and third trimesters of pregnancy is effective to prevent perinatal transmission of HBV^12–14^. Unfortunately, some infants born to mothers undergoing antiviral therapy during the second or third trimester of pregnancy were infected with HBV^13,15^. As a result, the most effective time to start an antiviral treatment is unknown. Our results showed that for pregnant women with active chronic hepatitis B (CHB), continuous antiviral treatment from preconception through the entire pregnancy is safe for both mothers and infants, and an effective means to reduce HBV entering offspring’s body.

In our study, we focused on the active CHB patients and chronic HBV carriers with high HBV DNA level, as they all should be given antiviral therapy to control hepatitis flare and reduce MTCT of HBV. Compared with Group C or Group D (antiviral therapy administrated in late pregnancy), other two groups (Group A, antiviral therapy from preconception; Group B, antiviral therapy in early pregnancy) did not present with significant maternal and infants’ complications (*P* > 0.05). Our results indicate that taking TDF/LDT therapy could be safe for the mothers and their offspring from preconception through the entire pregnancy. Although our study sample sizes were relatively small, those pregnant women had comparable characteristics (e.g., small age difference and unique Chinese ethnicity), and we used consistent laboratory test methods throughout the study. In the future study, more pregnant women may be needed to determine whether there may be possible effect of antiviral drugs on the congenital abnormality or birth defect. Furthermore, because the following-ups only were carried out within 28–36 weeks postpartum in previous studies^13,21,22^, our evaluation on the safety might be incomplete, thus the follow-up period needs to be extended.

Continuous antiviral treatment from preconception though the entire pregnancy is very important for active CHB patients to keep liver function normal and control maternal liver damage^23^. The cessation of antiviral therapy could result in severe exacerbations, even liver failure for CHB patients^24^. Compared with non-pregnant women, pregnant women are more susceptible to liver failure due to chronic HBV infection, because HBV infection is the most important causes of severe hepatitis during pregnancy in China^25^. ALT levels and HBV DNA load levels could be enhanced during the entire pregnancy, because the alterations immune regulation could contribute to the increase of HBV replication and ALT level during pregnancy^26–28^. Furthermore, HBV and ALT flares could even be severe during pregnancy^29^. CHB patients are liable to develop into liver fibrosis, even cirrhosis if their ALT flares are repeated and severe^30^. As a result, some pregnant women with CHB could develop into severe liver fibrosis, cirrhosis during pregnancy. In our study Group A (antiviral therapy from preconception), patients presented with normal ALT throughout pregnancy might be due to initiation of antiviral treatment during preconception and continuation of the regimen through the entire pregnancy. In our Group B and Group C, these patients’ liver function gradually turned to be normal after undergoing antiviral therapy. None of the patients developed into liver failure or cirrhosis. However, one patient in Group B and one in Group C were both excluded due to their own drug withdrawal. The two patients showed normal ALT while they took antiviral drugs. The ALT was 544 U/liter and 422.2 U/liter, respectively, after their withdrawing antiviral drug. However, ALT level gradually returned to normal after they took antiviral drug again.

Continuous antiviral treatment starting in preconception appears to be an effective means to control maternal hepatic damage and reduce HBV transmission from mother to the fetus. Compared with maternal HBV DNA level and ALT level of other groups at delivery (Table 1), Group A patients (antiviral therapy from preconception) were with lower HBV DNA level and normal ALT throughout the pregnancy, showing that continuous antiviral treatment from preconception through the entire pregnancy effectively have reduced maternal HBV DNA level and hepatic damage. The lower maternal HBV DNA level was, the lower the risk of HBV entering offspring’s body was. The lower the risk of HBV entering offspring’s body was, the higher the success rate of infant immunoprophylaxis was. Additionally, in the present study, none of infants that completed follow-up showed HBsAg positive, and all infants showed HBsAb positive. None of infants in our study was infected with HBV. It is generally accepted that maternal HBV DNA level plays a vital role in MTCT of HBV^4,9–11^. Several recent studies^13,14,21^ reported that MTCT rate of HBV was reduced if patients took antiviral therapy (e.g., LAM, LDT and TDF) during the late pregnancy. Our results also demonstrated that antiviral therapy during pregnancy decreased MTCT rate of HBV. Moreover, in our study, some infants with HBcAb positive, HBsAb positive and HBsAg negative indicated that even if MTCT of HBV did not occur, HBV still entered some offsprings’ body. Because the HBcAb positive rate at age of 0–24 months might partly result from the passive transfer of their mothers’ body or mean that HBV has ever entered into infants’ body^31,32^, we would further study the sense of HBcAb to infants.

In previous studies^13,22^, antiviral therapy continued to 1 month postpartum to interrupt MTCT of HBV. In our study, patients with high HBV DNA level and normal liver function during pregnancy immediately discontinued antiviral therapy after delivery. Then these mothers could breast-feed their infants. Although EASL guideline in 2017^19^ recommended that patients who continued TDF therapy postpartum should be advised to breast-feed, most mothers who continued TDF therapy after delivery remained unwilling to breast-feed because they were afraid of any potential adverse effect of TDF on their infants in our clinical practice. Furthermore, TDF drug labels, the clinical guidelines of the Chinese Society of Hepatology^33^, and the clinical guidelines of Asian Pacific^34^ do not clearly recommend that breastfeeding be encouraged while the mother undergoes TDF treatment.

In summary, our findings suggest that continuous antiviral treatment from preconception through the entire pregnancy is safe for active CHB mothers and their infants. This effective means can reduce HBV entering offspring’s body. Continuous antiviral therapy throughout pregnancy can prevent MTCT of HBV. Furthermore, antiviral therapy appears to control hepatitis flare and promote ALT normalization during pregnancy. Initiation of antiviral therapy for prospective pregnant women with active CHB may be advanced to preconception.

## Methods

### Patients

The current prospective cohort study was approved by the institutional ethics review committee of the Third Affiliated Hospital of Guangzhou Medical University, and was carried out in accordance with the ethical standards laid down in the 1964 Declaration of Helsinki and its later amendments. Informed written consent was obtained from each patient in the present study. The present study was registered in ClinicalTrials.gov (No. NCT03181607). We had access to the study data and had reviewed and approved the final manuscript.

This multicenter cohort study was conducted between January 2017 and December 2018. Pregnant women with chronic HBV infection, who visited six hospitals in China and met the following inclusion criteria and exclusion criteria, were enrolled on a voluntary basis. The inclusion criteria were as follows: (a) a history of HBV infection ≥ 6 months; (b) positive for HBsAg; (c) for patients already administrated with antiviral treatment in preconceptional period, the therapy could not be discontinued; for patients never administrated with antiviral treatment, ALT ≥ 2 times the upper limit of normal (ULN), or HBV DNA level ≥ 10^4^ IU/ml (positive for HBeAg) or HBV DNA level ≥ 10^3^ IU/ml (negative for HBeAg), traditional protecting liver and reducing enzyme treatment failed; (d) good compliance of patients.

The exclusion criteria were as follows: (a) patients with antibodies against HIV, HCV, HDV, or other forms of chronic liver disease; (b) evidence of hepatocellular carcinoma, decompensated liver disease, auto-immune hepatitis, or significant renal, cardiovascular, respiratory or neurological comorbidity; (c) concurrent treatment with nephrotoxic drugs, glucocorticoids, cytotoxic drugs, non-steroidal anti-inflammatory drugs, or immune modulators; (d) ultra-sonographic evidence of fetal malformation, abnormal fetal development or placental abnormality; (e) clinical signs of threatened miscarriage; (f) a history of complicated pregnancy; (g) drug discontinuance by themselves.

### Study design

As shown in Fig. 1, according to the inclusion criteria and exclusion criteria, 136 eligible pregnancy women with chronic HBV infection were enrolled in our study. They were assigned to the following four groups. In group A, patients with active CHB received an oral dose of 300 mg of tenofovir disoproxil fumarate (TDF, GlaxoSmithKline) or 600 mg of telbivudine (LDT, Novartis China) once per day due to ever abnormal liver function. When their liver enzyme turned to be normal prior to pregnancy, they took TDF or LDT continuously during their entire pregnancy. In group B and group C, pregnancies with CHB showed abnormal liver function in early pregnancy (less than 24 gestational weeks) and in late pregnancy (more than 24 gestational weeks), respectively. Then they began to take TDF (300 mg/times) or LDT (600 mg/times) every day, respectively. In group D, the pregnant women showed high HBV DNA level (≥ 10^6^ IU/ml) and normal liver function. To reduce HBV DNA level and risk of MTCT, they were treated with TDF (orally 300 mg/times, Qd) or LDT (orally 600 mg/times, Qd) during the late pregnancy.

During pregnancy, all patients were followed up every 4 weeks to monitor adverse events and laboratory results (liver function tests, HBV DNA level, hematologic tests, etc.). Following delivery, patients in Groups A, B and C continued to receive TDF/LDT therapy, and patients in Group D stopped receiving TDF/LDT therapy. All these patients were also followed up every 4 weeks in the first 3 months of delivery. They subsequently were followed up at 24th week and 28th week postpartum, respectively.

All infants received 100 IU of hepatitis B immune globulin (HBIG, Hualan Biotechnology Co., Ltd. Xinxiang City, Henan Province, China) and 10 μg of the recombined hepatitis B vaccine (RHBV, Shenzhen Kangtai Biotechnology Co., Ltd., Shenzhen City, Guangdong Province, China) intramuscularly within 1–24 h after birth. The same dose of HBIG was administrated at age of 4 weeks. The same dose of recombined hepatitis B vaccine was given at week 4 and week 24.

All mothers and their infants were followed up to 7 months postpartum.

### Outcome measures

The primary outcome included the efficacy and the safety of antiviral therapy for mothers. We analyzed maternal HBV DNA level and alanine transaminase (ALT) at delivery and 7 months postpartum. To measure the safety of antiviral therapy on the mothers, adverse complications of mothers were analyzed.

The secondary outcome included the effectiveness of blocking MTCT of HBV and the safety of TDF/LDT for the offspring. Fetal development indicators such as birth weight, body length and congenital abnormalities at birth and at age of 6 months were evaluated to assess the safety of antiviral therapy with respect to fetal complications. HBV antigen and antibody status of infants were assayed at age of 7 months to assess the effectiveness of blocking MTCT of HBV.

### Statistical analyses

All data were analyzed using SPSS v22.00 statistical analysis software (SPSS Inc, Chicago, IL). Descriptive variables were expressed as mean ± standard deviation (SD) or percentage. Analysis of variance (ANOVA) test and *χ2* test were used to compare quantitative and categorical variables, respectively. Differences were considered statistically significant at a *P* value < 0.05.

## Conclusion

Our findings from this clinical trial apparently support our hypothesis that continuous antiviral treatment from preconception through the entire pregnancy is safe for active CHB mothers and their infants. Antiviral therapy appears to control hepatitis flare and promote ALT normalization during pregnancy. Initiation of antiviral therapy for prospective pregnant women with active CHB may be advanced to preconception.
